# Pharmacokinetics of apixaban in patients undergoing pancreaticoduodenectomy (PAP-UP) 

**DOI:** 10.5414/CP204502

**Published:** 2024-04-25

**Authors:** Richard Zheng, Edwin Lam, Peter Altshuler, Madison Crutcher, Harish Lavu, Charles J. Yeo, Douglas Stickle, Benjamin Leiby, Walter K. Kraft

**Affiliations:** 1 Department of Surgery, Johns Hopkins Hospital, Baltimore, MD,; 2 Janssen Research and Development, Johnson & Johnson, Spring House,; 3 Department of Surgery,; 4 Department of Pathology, and; 5 Department of Pharmacology, Physiology and Cancer Biology, Thomas Jefferson University, Philadelphia, PA, USA

**Keywords:** pancreaticoduodenectomy, apixaban, pharmacokinetics, bioequivalence

## Abstract

Objective: The impact of pancreaticoduodenectomy on absorption of drugs in the duodenum remains largely unknown. We aim to characterize the pharmacokinetics of apixaban in patients who had previously undergone pancreaticoduodenectomy. Materials and methods: A single 10-mg dose of apixaban was administered to 4 volunteers who underwent pancreaticoduodenectomy at least 6 months prior. The maximum plasma apixaban concentration (C_max_) and area under the plasma concentration time-curve (AUC_0–24,_ AUC_0–inf_) were compared against healthy historical control subjects (N = 12). Geometric mean ratios (GMR) with 90% confidence interval (CI) were calculated for determination of comparative bioequivalence. Results: In pancreaticoduodenectomy patients, AUC_0–24_ and AUC_0–inf_ were 1,861 and 2,080 ng×h/mL, respectively. The GMRs of AUC_0–24_ and AUC_0–inf_ between study subjects and healthy controls were 1.27 (90% CI 0.88 – 1.83) and 1.18 (90% CI 0.82 – 1.72). The mean C_max_ of apixaban was 201 ng/mL (SD 15.6) occurring at a median t_max_ of 3.25 hours (range 2.5 – 4 hours). The GMR of C_max_ between study subjects and healthy controls was 1.12 (90% CI 0.77 – 1.63). Conclusion: The pharmacokinetic characteristics of apixaban in subjects who had undergone pancreaticoduodenectomy are not significantly different from those of healthy controls. Though the sample size of this study is small, results suggest that no change to apixaban dose regimen is needed in patients who have had a pancreaticoduodenectomy.

**What is known about this subject**

- Apixaban is absorbed in the duodenum and proximal small bowel but little is known regarding the absorption kinetic profiles after pancreaticoduodenectomy (Whipple procedure), during which the duodenum and head of pancreas are removed.

**What this study adds**

- This study suggests that pancreaticoduodenectomy may have no clinically significant effect on the metabolism of apixaban.

## Introduction 

Pancreaticoduodenectomy, also known as the Whipple procedure, is a surgery in which most or all of the duodenum as well as the head, neck, and uncinate process of the pancreas are resected. This leads to changes in gut physiology and metabolism, including a reduction in gastrointestinal hormone secretion, changes in first pass metabolism, delayed gastric emptying, and selective vitamin and mineral deficiencies [1, 2, 3, 4, 5, 6]. Additionally, the duodenum and proximal jejunum are the major sites of absorption for many drugs due to their microvilli and correspondingly large surface area [7]. With pancreatic cancer outcomes improving, incidence increasing, and projections that pancreatic cancer will become the second leading cause of cancer-related death in the United States in the next several decades, we will likely be managing the long-term health conditions of pancreaticoduodenectomy survivors for years to come [8]. 

Apixaban is a direct-acting oral anticoagulant (DOAC) with labeled indication for the treatment of venous thrombosis. Patients undergoing major gastrointestinal surgery are at significantly elevated risk of developing thrombotic complications and are not infrequently treated with apixaban. Apixaban is primarily absorbed in the proximal small bowel, i.e., the duodenum and jejunum [9]. Studies of luminal apixaban dosing have shown that apixaban exposure decreases as the drug is delivered more distally in the gastrointestinal tract, with a 60% reduction in exposure when apixaban is delivered directly to the distal ileum as opposed to orally ingested, and an even greater reduction of absorption when delivered to the colon [10]. Most or all of the duodenum and a 20- to 30-cm segment of proximal jejunum are removed during pancreaticoduodenectomy, which theoretically could lead to a decrease in systemic drug exposure. However, the effect of pancreaticoduodenectomy on apixaban delivery has never been studied. 

We hypothesized that patients who undergo pancreaticoduodenectomy have a geometric mean ratio (GMR) of drug exposure (AUC_(0–24)_) and maximum concentration (C_max_) less than the apixaban AUC_(0–24)_ and C_max_ of healthy subjects who had not undergone pancreaticoduodenectomy. 

## Materials and methods 

### Study design 

**Figure 1.**
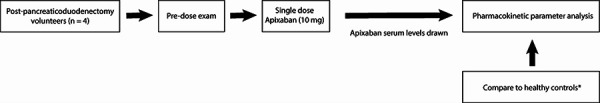
Study design. *Data for healthy controls were obtained from a separate study also involving administration of 10 mg apixaban to 12 volunteers and subsequent pharmacokinetic parameter analysis [13].

This was a phase I, investigator-initiated, open-label, single-arm, single-site, single-sequence trial performed in volunteers at the Clinical Research Unit of the Thomas Jefferson University Hospital (TJUH). Subjects were given a single 10-mg dose of apixaban under fasting conditions. Blood was drawn at 0, 0.5, 1.5, 2.5, 4, 6, 10, and 24 hours post dose through an indwelling catheter. A schematic of the study design is shown in Figure 1. 

The primary endpoints were GMR of maximum concentration (C_max_) and area under the time-concentration curve (AUC_0–24_, AUC_0–inf_), defined as the ratio of geometric means between the two groups of experimental subjects and historical controls. Formal bioequivalence is defined by the Food and Drug Administration (FDA) as the 90% confidence interval (CI) for those aforementioned values being contained within an 80 – 125% interval of established norms from historical controls, and these were the bounds used to determine bioequivalence in this study [11]. 

### Subjects 

Participants in this study must have undergone a pancreaticoduodenectomy over 6 months prior to study enrollment, be otherwise healthy men or women between the ages of 18 and 65 (inclusive) and be willing to comply with trial restrictions for the period of the study. Subjects were excluded from the study if they had any ongoing clinically significant medical conditions or evidence of active malignancy, experienced any recent major bleeding event or major surgery within 6 months of enrollment, were actively smoking tobacco or using other illicit drugs, were pregnant or planning to become pregnant, or had any evidence of increased bleeding risk at the discretion of study investigators. The routine use of strong cytochrome P450 inhibitors (CYP3A4) or dependence on oral pancreatic enzyme replacement for digestion after surgery also excluded patients from enrollment. This study was approved by the Thomas Jefferson University Institutional Review Board and all subjects provided written informed consent. 

### Pharmacokinetic analysis 

Whole blood for apixaban pharmacokinetic analysis was collected into 4-mL dipotassium EDTA tubes and immediately subjected to plasma separation by centrifugation at 1,000 g for 15 minutes. Once separated, plasma was stored at –20°C until ready for processing. The plasma concentration of apixaban was measured by the Jefferson University Hospital clinical laboratory, using a liquid chromatography-tandem mass spectrometry (LC-MS/MS) assay validated for research use only [12]. d4-rivaroxaban was used as the internal standard. The calibration curve in plasma was linear over the range of 6.0 – 600 ng/mL. The between-run precisions for all levels of quality control samples was below 10% coefficient of variation. 

Single-dose pharmacokinetic parameters were determined based on plasma concentrations over time. Maximum plasma concentration (C_max_) and time to maximum concentration (t_max_) were calculated. Individual pharmacokinetic parameters will be determined by non-compartmental methods using PKAnalix software (v. 2021, Lixoft). Area under the time-concentration curve (AUC_0–24_ and AUC_0–∞_) were calculated using the linear up-log down method. Plasma half-life (T_1/2_) was estimated as log(In)2/λz, in which the slope of the terminal phase of the plasma concentration-time curve was determined by the least squares method (log linear regression of at least three data points) with a weighting factor of 1. 

### Historical controls 

The historical control data for this study were obtained from a prior study from our institution, which was a phase I, investigator-initiated, open-label, randomized, two-sequence study with co-administration of apixaban and either tacrolimus or cyclosporine to healthy volunteers using the same analytic method in this study [13]. The data taken was limited to period I of the study, during which 12 subjects received a single 10-mg dose of apixaban alone. Serum levels were collected immediately before and at 1, 2, 3, 4, 6, 12, 24, 48, and 72 hours after apixaban administration; however, data from the 48- and 72-hour timepoints were not included in the analysis to make the sampling endpoints equivalent between historical controls and study subjects. 

### Statistical analysis 

The target sample size of 6 subjects was determined on a pragmatic basis for the purpose of an exploratory study. There was no formal power analysis. GMRs for AUC and C_max_ were generated by first log-transforming values for those parameters. We then performed a two-sample t-test with a confidence level of 90% on these log-transformed values, and the differences between means were back-transformed to generate GMRs and their corresponding 90% CI bounds. C_max_, volume of distribution, and clearance were reported as arithmetic means with standard deviation. t_max_ was reported as a median value with a corresponding range of values. All statistical analyses were performed with Stata v 13.0 (StataCorp LLC, College Station, TX, USA). 

## Results 

### Demographics 

Due to the onset of the COVID-19 pandemic coinciding with the rollout of this study and the logistical challenges of accruing volunteers in that context, we enrolled 4 subjects in total. All 4 enrolled subjects completed the study. Demographic data regarding age, race, body mass index, pathology, creatinine clearance, and time from pancreaticoduodenectomy to study enrollment are summarized and compared with historical controls in Table 1. All subjects in the study had a history of pylorus-preserving pancreaticoduodenectomy. There were no adverse events recorded during this study. 

### Pharmacokinetics 

**Figure 2.**
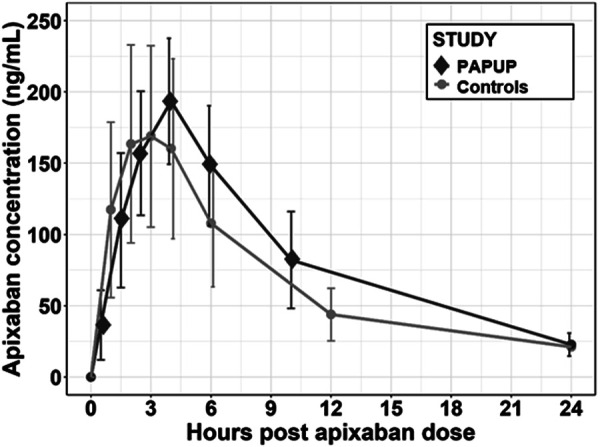
Mean apixaban plasma drug concentration-time profiles of post-pancreaticoduodenectomy subjects compared against historical controls. Mean plasma concentration-time profiles of apixaban in 4 post-pancreaticoduodenectomy subjects (PAP-UP) or in 12 healthy historical controls following a single 10-mg oral dose of apixaban alone. Each dot represents a time point where serum apixaban levels were measured. PAP-UP: pharmacokinetics of apixaban in patients undergoing pancreaticoduodenectomy.

Pharmacokinetic parameters (AUC, maximum concentration, time to maximum concentration, half-life, volume of distribution, and clearance) for the 4 study subjects and 12 historical controls are enumerated in Table 2. Aggregated study data versus healthy controls are plotted in Figure 2. 

### Bioequivalence (geometric mean ratios) 

The GMR of AUC_0–24_ and AUC_0–inf_ between study subjects and healthy controls were 1.27 (90%CI 0.88 – 1.83) and 1.18 (90% CI 0.82 – 1.72), respectively. The GMR of C_max_ between these same populations was 1.12 (90% CI 0.77 – 1.63). 

## Discussion 

In this limited sample of subjects who had undergone pancreaticoduodenectomy, the absorption and metabolism of apixaban are similar to that of healthy volunteers. Although our results did not meet formal FDA bioequivalence criteria, these criteria are typically used to determine strict bioequivalence for generic drugs. In a more practical context, we observe that the clinical bounds for the 95% CI may be wider than that for generic drug bioequivalence. Indeed, the FDA-approved apixaban product label recommends no apixaban dosage adjustments even with the coadministration of strong CYP3A4 inhibitors such as diltiazem, even though its coadministration may increase serum apixaban levels 1.5- to 2-fold [14]. In all, our findings suggest that pancreaticoduodenectomy leads to a clinically insignificant difference in pharmacokinetic parameters that should not immediately change apixaban dosing in these patients. However, the trends observed in this limited cohort – that t_max_, C_max_, and AUC were slightly increased in patients who had undergone a pancreaticoduodenectomy – could still be clinically relevant and apixaban dosing in this population should still be monitored with caution. 

The lack of difference in absorption after pancreaticoduodenectomy points to the large resorptive capacity of the jejunum and ileum after resection of the duodenum. It has previously been demonstrated that the majority of absorption for drugs subject to cytochrome P450 3A (CYP3A)-mediated first-pass metabolism such as apixaban occurs in the proximal small bowel, where CYP3A4 is most heavily expressed [15]. The duodenum also has relatively more and larger villi for absorption of substrate [16]. Despite this, however, the distal small bowel and colon are able to contribute up to 55% of apixaban absorption [17], implying that they are capable of increasing absorption when the duodenum is partially or totally removed. 

In these patients, this change in absorption seemingly occurs despite the decrease in exocrine function that is expected after pancreaticoduodenectomy [18]. Over 50% of pancreaticoduodenectomy patients have experienced some degree of postoperative pancreatic exocrine insufficiency. As such, they are commonly dependent on oral pancreatic enzyme replacement therapy (i.e., pancrelipase) – in our study, we find that absorption of apixaban remains similar to those of non-surgical controls even in the absence of these enzymatic replacements. 

When compared to other pharmacokinetic studies utilizing an equivalent 10-mg dose of apixaban, we also find that our results largely fit within the same range of absorption parameters in other populations. Our mean C_max_ of 201 ng/mL lies in a range of 144 – 287 ng/mL, with the highest C_max_ found in healthy subjects who had ingested apixaban emulsified into an oral solution [19]. Similarly, most studies have also estimated t_max_ to be 3 hours; crushed tablets led to slightly faster absorption (2 hours) [20], whereas morbidly obese subjects or subjects with renal failure had slightly delayed absorption (4 hours) [21, 22]. The total exposure to apixaban in pancreaticoduodenectomy patients is also consistent with other published studies, although T_1/2_ in our study (6.8 hours) was the shortest among such studies (range 8.1 – 17.3 hours). This difference may be an artifact of the plasma collection schedule in our study, wherein collection times were densely clustered within the first few hours but did not extend beyond 24 hours post dose. 

Pharmacokinetic studies of this unique subpopulation of pancreaticoduodenectomy patients have thus far to date been limited to a single study, wherein patients who had a pancreaticoduodenectomy were demonstrated to have a serum-drug concentration curve of acetaminophen similar to those of normal controls [23]. Although data on pharmacokinetics after pancreaticoduodenectomy are scarce, drug absorption and metabolism have been more extensively studied in patients undergoing gastric bypass surgery. This population has similarly altered gastrointestinal anatomy akin to the group in our study, albeit with much longer segment of intestine (typically 100 – 150 cm) that is bypassed. A study of the absorption of DOACs in post-bariatric surgery subjects demonstrated that peak serum levels of apixaban and dabigatran were within the normal expected range after bariatric surgery, although peak rivaroxaban levels were significantly lower compared to matched individuals who did not undergo bariatric surgery [24]. In all, these studies reinforce the idea that the distal small bowel is generally able to increase drug absorption when the proximal small bowel is surgically diverted, but that even different medications within the same class should be looked at carefully. 

The statistical validity of our study is significantly limited by a small sample size and thus should be considered an exploratory investigation. The advent of the COVID-19 pandemic during the initial recruitment phase of the study significantly limited the ability to recruit volunteers for a non-therapeutic drug trial. Similarly, although the 12 historical controls used in this study are also few in number, this comparator group was specifically selected because their apixaban dosing was identical and the serum collection schedule was similar. While the total number of subjects was less than planned for due to COVID-19, the sampling density was very dense particularly in the early part of the curve representing absorption. In addition, AUC_0–24_ represented ~ 90% of AUC_0–∞_ with the extrapolated fraction well below the generally accepted maximum of 25%. Lastly, subjects in this study were all several years from their initial surgery (mean 56 months), so the findings from this study may not be applicable to patients who are in the early phase of recovery after pancreaticoduodenectomy, before the jejunum and ileum adjust to the absence of the duodenum. 

## Conclusion 

The pharmacokinetic parameters of apixaban in pancreaticoduodenectomy patients described in this study show clinically insignificant differences from healthy controls. Based upon the results, the current clinical approach of using FDA labeled dosing in pancreaticoduodenectomy patients is warranted. There is no evidence to suggest the need for dose modification of apixaban in this population, but further study in a larger population is warranted. Although obtaining large-scale pharmacokinetic data may not be feasible in this specific population of patients undergoing pancreaticoduodenectomy, retrospective data on the use of apixaban may help to corroborate the findings of this preliminary study. Although limited by small sample size, the lack of significant changes in pharmacokinetics seen in pancreaticoduodenectomy patients relative to healthy controls in this study suggest that standard apixaban dosing is appropriate in the post-pancreaticoduodenectomy population. 

## Acknowledgment 

We would like to recognize and thank Angela Pallotto, Courtenay Fulmor, and Lisa Pullaro, our staff in the Clinical Research Unit, and the Saligman family for providing funding to the Jefferson Department of Surgery. 

## Study registration 

ClinicalTrials.gov Identifier: NCT04191928. 

## Authors’ contributions 

Study conception and design: Richard Zheng, Harish Lavu, Charles Yeo, Douglas Stickle, Benjamin Leiby, Walter Kraft. 

Acquisition of data: Richard Zheng, Peter Altshuler, Madison Crutcher, Douglas Stickle, Walter Kraft. 

Analysis and interpretation of data: Richard Zheng, Edwin Lam, Benjamin Leiby, Walter Kraft. 

Drafting of manuscript: Richard Zheng, Walter Kraft. 

Critical revision of manuscript: Richard Zheng, Edwin Lam, Peter Altshuler, Madison Crutcher, Harish Lavu, Charles Yeo, Douglas Stickle, Benjamin Leiby, Walter Kraft. 

## Funding 

Funding support was provided by the Saligman Family pilot grant, which was made available to study investigators by the Thomas Jefferson University Hospital Department of Surgery. Drs. Zheng, Crutcher, and Altshuler were supported by National Institutes of Health institutional training grant (T32GM008562). 

## Conflict of interest 

The authors of this study do not have any financial conflict of interest to disclose. 

**Table 1.** Subject demographics.

ID	Age (y)/ Gender	Race	BMI (kg/m^2^)	CrCl (ml/min)	Indication for surgery	Time from surgery (months)
PAP-UP						
101	55/M	White	22	92	Ductal adenocarcinoma	36
102	41/F	Asian	36	205	IPMN	73
103	61/F	White	21	123	MCN	61
104	62/M	White	38	80	Tubular adenoma	55
Mean	55	–	29	125	–	56
Historical controls						
201	48/M	Black	33.0	–	N/A	N/A
202	54/M	White	23.8	–	N/A	N/A
203	25/M	Black	27.2	–	N/A	N/A
204	30/M	Black	26.6	–	N/A	N/A
205	25/M	Black	29.1	–	N/A	N/A
206	29/M	Black	32.8	–	N/A	N/A
207	53/M	Black	27.0	–	N/A	N/A
208	52/M	Black	32.9	–	N/A	N/A
209	54/M	White	32.8	–	N/A	N/A
210	44/M	Black	26.3	–	N/A	N/A
211	42/M	Black	27.9	–	N/A	N/A
212	38/M	White	30.9	–	N/A	N/A
Mean	41	–	29.1	–	–	–

BMI = body mass index; CrCl = creatinine clearance calculated according to the Cockroft-Gault equation [25]; PAP-UP = pharmacokinetics of apixaban in patients undergoing pancreaticoduodenectomy; IPMN = intraductal papillary mucinous neoplasm; MCN = mucinous cystic neoplasm.

**Table 2.** Pharmacokinetic parameters of post-pancreaticoduodenectomy subjects compared against historical controls.

Parameter	Format	PAP-UP	Controls
AUC_0–24_ (ng×hr/mL)	Geometric mean	1861	1492
	GMR	1.27 (90% CI 0.88 – 1.83)	
AUC_0–inf_ (ng×hr/mL)	Geometric mean	2080	1757
	GMR	1.18 (90% CI 0.82 – 1.72)	
C_max_ (ng/mL)	Geometric mean	201	179
	GMR	1.12 (90% CI 0.77 – 1.63)	
t_max_ (h)	Median	3.25	2.5
T_1/2_ (h)	Arithmetic mean	6.8	8.4
V_d_ (L)	Arithmetic mean	47.9	63.8
Clearance (L/h)	Arithmetic mean	4.9	5.7

PAP-UP = pharmacokinetics of apixaban in patients undergoing pancreaticoduodenectomy; AUC = area under the curve; GMR = geometric mean ratio.
